# Assessing a community health center-driven process for engaging with translational scientists: What will it take?

**DOI:** 10.1017/cts.2025.10058

**Published:** 2025-06-09

**Authors:** Tracy A. Battaglia, Kareem I. King, Allyson Richmond, Erika Christenson, Rebecca Lobb, Astraea Augsberger, Celia Bora, Stephen M. Tringale, Linda Sprague Martinez, Charles T. Williams

**Affiliations:** 1 Boston University School of Medicine, Clinical and Translational Science Institute, Boston, MA, USA; 2 Section of General Internal Medicine, Department of Medicine, Boston Medical Center and Boston University Chobanian & Avedisian School of Medicine, Boston, MA, USA; 3 Yale Cancer Center, Yale School of Medicine, New Haven, CT, USA; 4 Boston HealthNet, Boston, MA, USA; 5 Boston University School of Social Work, Boston, MA, USA; 6 NeighborHealth, formerly East Boston Neighborhood Health Center, Boston, MA, USA; 7 Codman Square Health Center, Dorchester, MA, USA; 8 Health Disparities Institute, University of Connecticut School of Medicine, UConn Health, Hartford, CT, USA; 9 Department of Family Medicine, Boston Medical Center, Boston, MA, USA

**Keywords:** Community health center, partnership, translational science, community engagement, health equity, translational research, toolkit

## Abstract

Actively engaging community health centers (CHCs) in research is necessary to ensure evidence-based practices are relevant to all communities and get us closer to closing the health equity gap. We report here on the Boston HealthNet Research Collaborative, a partnership between health centers, Boston HealthNet and the Boston University Clinical, and Translational Science Institute with the explicit goal of supporting research partnerships early in the planning phase of the study lifecycle. We used the principles of community engagement guided by a collective impact framework to codesign, pilot, and evaluate a process for facilitating research partnerships. Accomplishments in the first 2 years include a web-based Toolkit with a step-by-step guide and an active learning collaborative with health center representatives to support research capacity building. The process resulted in 81 new research project partnerships across 50 individual research projects. Most research partnership requests were made later in the research lifecycle, after the planning phase. Partnership acceptance was largely driven by the Collaborative’s pre-defined *Guiding Principles* and *Rules of Engagement.* These lessons drive an iterative process to improve the longitudinal relationship between our translational research program and our CHC partners.

## Background

Community health centers (CHCs) are nonprofit, patient-governed organizations that deliver high-quality, comprehensive primary healthcare to America’s economically disadvantaged populations [1]. Serving 1 in 11 people nationwide, CHC’s served 32.5 million patients in 2021 [2], the majority of whom are low income, members of minoritized populations, and publicly insured or uninsured. Given their historic roots in both healthcare access and community relations, CHCs are at the forefront of addressing health inequity in our nation [3,4]. The ability of CHCs to close the health equity gap is dependent on the availability of evidence-based practices that are relevant to the communities that they serve. Unfortunately, it is well-recognized that CHCs and the communities they serve remain underrepresented in research. This poses a translational research barrier that contributes to health inequity [5].

Evidence suggests a disconnect between CHC’s interest in research and their actual participation. A national survey of federally qualified health centers for example found that while the majority were interested in research only about half actively participate in research-related activities, and when they do, the research is rarely led or initiated by the health center [6]. Lack of dedicated staff time and concern about loss of productivity or income were the most commonly cited barriers to participation, followed by lack of training or knowledge in conducting research and a lack of policy or infrastructure to carry out research on site [7]. Surveys in Massachusetts and South Carolina CHCs documented similar barriers to research participation with the authors concluding that CHCs are neither resourced to participate nor do systems empower them to participate in research [8,9]. Successful models that foster bidirectional and longitudinal community–academic relationships are necessary to address these barriers to CHC-driven research [10].

Boston HealthNet (BHN) is a network affiliate of Boston Medical Center (BMC), Boston University School of Medicine (BUSM), and 13 CHCs delivering care to the city’s racially and ethnically diverse and working-class neighborhoods. This integrated healthcare delivery system provides outreach, prevention, primary care, behavioral health, specialty care, and dental services at sites located throughout Boston. Because of a longstanding commitment to research that improves the health of the communities they serve, the BHN came together with the Boston University Clinical and Translational Science Institute (BU CTSI) back in 2005 to codevelop a process with their Institutional Review Board to ensure no research was happening in the CHCs without their knowledge. Using a collective impact approach [11], a written consent process put CHC leadership in control of all research projects entering their health center. With the COVID pandemic came an unprecedented opportunity to offer access to several time-sensitive clinical trials across our CHC network. This was the catalyst for both CHC leaders and researchers to recognize that while necessary, this one-time consent process was not sufficient to facilitate equitable research partnerships that reflect community priorities. Therefore, in 2021, the BHN and the BU CTSI partnered to establish the *Boston HealthNet Research Collaborative* to overcome the lack of health center engagement in research. We report here our progress with the explicit goal of supporting research partnerships early in the planning phase of the study lifecycle.

## Approach

We used the principles of community engagement guided by the collective impact framework to design, pilot, and evaluate a process for facilitating research partnerships with a shared goal of connecting researchers with CHC partners during the planning phase of the research [12,13]. This iterative process is supported by the BU CTSI Community Engagement Program and driven by the following engagement principles: a shared governance structure, cocreation, bidirectional training and education, shared financing, and long-term commitment [14].

### Shared Governance and Co-Creation

A steering committee, comprised of faculty and staff leaders of both BHN and CTSI, formed in 2021 to launch the *BHN Research Collaborative*. First, we administered a short survey to CEOs at all BHN-affiliated CHCs to determine what they want from research partnerships. At the time, there were 11 health centers in BHN. Several themes emerged and formed the basis for discussion during planning meetings. CHCs desired:

1. Alignment with community needs: research that addressed community priorities and benefited all stakeholders including patients, staff, boards, and communities.
2. Early Engagement: to accurately budget and plan for a research project’s resource needs.
3. Data Use: input on how their data would be used.
4. Information Sharing: results from their participation.
5. Resource allocation: to adequately support expectations from research projects.

Each CHC was invited to nominate representatives to join a series of four virtual 90-minute meetings during which participants discussed approaches to overcome these issues. Nine CHCs participated. The first meeting focused on cocreating a Memorandum of Understanding (MOU) which clearly articulates a shared agenda for the *BHN Research Collaborative* with roles and responsibilities for members. As previously published, the MOU emphasized research based on local health priorities, open communication, trusting relationships, equity and building capacity for research at the CHC [13]. Subsequent meetings focused on codesigning a web-based Toolkit for researchers interested in partnering with CHCs [15].

CHCs unanimously agreed that having a designated Research Liaison (RL) on site at their CHC was desirable (versus a centralized model at the academic institution). A job description was cocreated outlining key activities and criteria for an RL: 1) be the point-person for all incoming research inquiries, with access to leadership for decision-making and 2) participate in a monthly virtual learning collaborative to gain relevant skills in research administration and share best practices with other sites. A clinical background was recommended but not required. Recruitment of the RL was left to the discretion of the CHCs, with the expectation that up to 4 hours per month may be required for the role. Nine of the CHCs designated an RL; five were clinical providers (physicians or nurse practitioners), while the others were practice staff representing population health or quality improvement. The two remaining sites declined to participate in the collaborative, due to lack of interest or capacity constraints.

**Figure 1.**
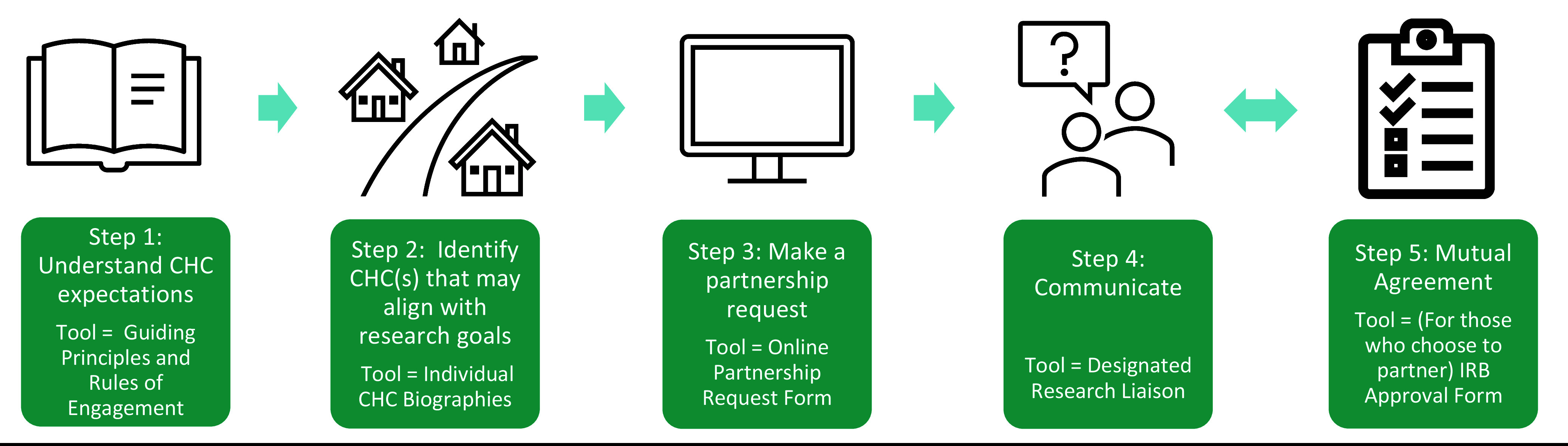
BHN partnership toolkit: a step-by-step guide for translational researchers who wish to conduct research *with* (not in) community health centers.

As shown in Figure 1, the Toolkit was designed as a step-by-step guide to directly respond to known partnership challenges and provide translational scientists tools to facilitate partnership development:

1. Understand CHC expectations: The researcher is encouraged to consult the *Guiding Principles and Rules of Engagement* document that describes the four founding principles that CHCs require to engage in partnership with a researcher (Research and Health Center Priority Alignment, Regular Communication, Equitable Partnerships, Potential Benefits), with specific examples of activities for both the researcher and CHC to operationalize each principle.
2. Identify CHC site(s) that may align with research objectives: Researchers may identify CHCs with potential alignment utilizing the *CHC Biographies*. These one-page summaries include practice information, patient demographics, priority health topics, and contact information.
3. Make a formal research partnership request: Researchers complete a web-based *Research Partnership Request Form* to initiate a partnership. The 31-item Qualtrics survey includes discrete and open-ended responses to provide relevant information about the proposed research for CHCs to determine if a partnership is desirable and feasible. Researchers choose which individual CHC(s) on the platform receive the application.
4. Communicate: A designated CHC Research Liaison receives the *Research Partnership Request* Form via email and is asked to reply with a decision on whether to accept or decline the request within 2 weeks. The internal process for decision-making may vary by site and often involves bidirectional communication with the researcher to gather additional information.
5. Mutual agreement: Once the researcher and CHC agree on a partnership, they submit a signed *IRB Approval Form* which summarizes expectations for both partners at the time of IRB submission.

### Shared financing

To compensate staff for their time spent serving as a Research Liaison, each participating CHC received an annual stipend of $10,000. The resources to fund these positions were shared equally by the CTSI and BHN. CHCs could utilize funds at their discretion.

### Bidirectional training and education

To support capacity-building for research at the CHCs, research liaisons participated in a monthly virtual learning collaborative and cohosted by the BU CTSI and BHN. Meetings were used to share program data, discuss successes, challenges, and topics brought forth by participants with the goal of learning from each other and leveraging their collective experiences. Guest speakers were invited to provide subject–matter expertise for specific questions such as completing IRB applications, conducting quality improvement projects, the basics of clinical research, research finance (e.g. billing and budgeting), and recruitment strategies.

### Long-term commitment

As expressed in the MOU, the goal of the *BHN Research Collaborative* is to build capacity for CHCs to engage in research centered on the priorities of the people they serve. We recognize this is going to require a long-term and iterative process, so each year the MOU is revisited to renew commitments and adapt the process as needed to serve all partners.

## Program evaluation

Data collection, measures, and analyses: The web-based *BHN research partnership request form* was launched using Qualtrics software (Qualtrics, Provo, UT) in January of 2022. The researcher completes the 31-item survey, which includes discrete and open-ended responses. Key information collected in the survey included: study team information, point of contact, IRB number, project timeline, project summary, project budget, and other information health centers may need to decide if the research project is fit for partnership with them. The online portal also included a drop-down menu for researchers to self-select which health centers they want to partner with on their project and a description of why that center is a good fit. Once the survey is complete, the researcher’s responses would be emailed directly to the designated CHC Research Liaison(s) for the selected health center. More information on the survey can be found in supplemental figure 1.

One month after the original request form is submitted, research liaisons receive an electronic five question *follow up survey* to document whether the CHC accepted or declined the partnership and why.

Two questions from the *partnership request form* used discrete categories to measure the phase of the study lifecycle at the time of the partnership request: 1) Project status = planning submission, funding awarded/project not started, or funding awarded/project started and 2) Institutional Review Board (IRB) review status = not yet submitted, under review, or approved. We defined early engagement as 1) project status = planning submission and 2) IRB status = not yet submitted. Then, we used the *follow up survey* to measure partnership status of each request: whether they accepted or declined, on behalf of the CHC. The early engagement outcomes were jointly decided on by the BHN Research Collaborative.

Research requests submitted between January 2022 and December 2023 to our online Qualtrics portal were summarized in Excel using Descriptive statistics [16]. Main outcomes included the frequency of early engagement at the time of partnership request in each calendar year and partnership status at follow-up. Directed content analysis was used to summarize open-ended responses related to reasons for acceptance and/or declining partnership requests [17]. Two authors (TB, KK) did initial coding, and a third author (AA) supported adjudication for 19 discordant codes.

Program findings: Nine out of 11 BHN CHCs opted to participate in the BHN Research Collaborative by nominating a Research Liaison, contributing a CHC Biography and signing an MOU. Eight of nine CHCs are in Boston proper, and one is situated just south of the city. Collectively, these CHCs serve over 190,000 patients who are largely from racially, ethnically, and linguistically minoritized groups. The size, racial, ethnic, and immigrant status of each CHC is unique.

From January 2022 to December 2023, a total of 50 research projects were submitted to the *BHN Research Partnership Request Form* (26 in 2022 and 24 in 2023). Overall, 31 of the 50 unique research studies submitted (62%) resulted in at least one CHC partnership invitation. Among these 50 research projects, 252 individual CHC partnership requests were submitted, with a median of 28 requests per CHC (data not shown). The research studies were submitted by 48 unique researchers most with an appointment of Associate (*n* = 19) or Assistant Professor (*n* = 15). Most applicants reported a primary affiliation with the Medical Center or the University (29 and 15, respectively). Four applicants were from outside institutions.

Research topics included 15 different health areas with the most common being infectious diseases, mainly HIV (*n* = 9), hypertension (*n* = 8), maternal health (*n* = 6), and mental health (*n* = 5). About half had or were seeking NIH funding (*n* = 26), while others sought funding from alternative sources (foundations, industry).

Content analysis of the 2-month *follow-up survey* open responses revealed several themes related to reasons for acceptance and declining research requests. These themes and reflective quotes are displayed in Table 1. These themes aligned with the original barriers identified in our planning survey and reflected the rules of engagement created by the collaborative. The primary reasons that drove decision-making were alignment between the CHC clinical and research priorities, the potential for the study to directly impact the patient or provider population (e.g., through enhancing current service delivery or providing new patient services), and the provision of adequate resources to enable participation in the research study. CHCs also reported the importance of pre-existing or trustworthy relationships with the researcher submitting the partnership request and open communication as needed to reach an informed decision.

**Table 1.** CHC reasons for accepting and declining research requests (*n* = 105)

Theme	Definition	Frequency	Accept select quotes	Decline select quotes
Alignment with community and CHC	Refers to whether the research topic and/or study methods are a “good fit” with CHC and population served; how well the methods align with priorities relevant to the CHC and population served	*n* = 39 28 accept 11 decline	*“aligns well with our efforts to improve care for patients with hypertension, a significant issue for our community and important focus of third-party quality metrics.” “This research question is pertinent to our patient population.”*	*“Not relevant to our patient population.” “Population at xxx not a good fit for study.” “We did not believe we had enough patients to accommodate the research.”*
Benefit to patients and/or CHC staff	Refers to the direct benefit that the research approach/methods provide to participants, above and beyond existing services or standard of care	*n* = 22 16 accept 7 decline	*“This proposal offered our patients living with HIV access to an intensive smoking cessation program that is not currently offered at the health center.” “…will augment current efforts to improve care for high risk hypertensive pregnant patients.” “Our pediatric providers are interested in augmenting the care we offer for children with hearing impairments and other disabilities.”*	*“Patients enrolling in this study could potentially be excluded from receiving the robust ongoing care we offer patients through our addiction services clinic.” “It seems like the study participants would receive the benefits for a small amount of time then be left unable to access these resources.”*
Adequate resources	Refers to the feasibility of study methods relative to available CHC infrastructure or resources needed to implement protocol	*n* = 21 14 accept 7 decline	*“The request required no effort from our data analyst.” “…there is a willing project champion …; and there is funding support possible if this proposal is selected for award.” “Low level of resources needed from the organization and participation will have a low impact on our operations.”*	*“After initial conversations, it was found that this research request required too many health-center supplied resources for us to support.” “We do not have space available nor a resource who would be able to manage identifying r and arranging the rooms.”*
Relationship between CHC and researcher	Refers to the prior relationship that a researcher or research team had with a health center	*n* = 8 7 accept 1 decline	*“Previous familiarity with project” “Previously worked with PI….it was a great learning opportunity for our clinicians”*	*“Conflict of interest between PI and study”*
Bidirectional communication	Refers to how responsive researcher is to CHC queries for clarification and/or willingness to tailor study methods prior to the partnership decision	*n* = 13 2 accept 11 decline	*“We are in continued conversations with this team about this research.” “We are in touch with the team while they complete this element of their plan.”*	*“…we asked whether the flyer had been modified since the last discussion; did not hear back.” “Initially contacted primary contact to set up meeting, never heard back.” “I sent an email asking for more information after discussing with staff at xxx and never heard back.”*
Other		*n* = 2 0 accept 2 decline		*“Currently not interested in pursuing” “*Have previously rejected this and explained why then.”

*n* = the number of responses to the follow-up survey that were coded under a particular theme.

## Discussion

We provide 2 years of evaluation data on a CHC-driven process that was designed to overcome persistent barriers to research partnerships in our safety net medical system – namely engagement at advanced phases of the research lifecycle. A Toolkit with a step-by-step guide was cocreated with CHCs to support researcher-initiated partnerships with CHCs and included an active learning collaborative with CHC representatives to support research capacity-building at the CHC sites. The process resulted in 81 new research project partnerships across 50 individual research projects. Most research partnership requests were made later in the research lifecycle, after the planning phase. Partnership acceptance was largely driven by the Collaborative’s pre-defined *Guiding Principles* and *Rules of Engagement*, namely alignment with CHC priorities, where there were clear benefits to patients or the practice and adequate resources were available to support CHC participation. Trustworthiness and communication responsiveness on behalf of the researcher were also key determinants of successful partnerships, reflecting the well-established principles of community engagement. These lessons drive an iterative process to improve the longitudinal relationship between our translational research program and our CHC partners.

Our CTSI engagement initiative reported here provides a blueprint for other academic medical centers to consider as one path toward the long-term goal of addressing the underrepresentation of marginalized and minoritized communities in clinical trials and health research. This translational research barrier is increasingly recognized as an important contributor to the “data divide” that perpetuates health disparities [18]. As advances in diagnostics and therapeutics drive personalized care, populations like those served at CHCs lack comprehensive and high-quality health data; this hinders their access to personalized and data-driven care. Our collaborative approach, which centers on the principles of community engagement and collective impact, was designed specifically to engage CHC providers in research, as there is considerable evidence that clinician recommendations play a key role in supporting patients to consider participation in clinical trials [19]. Yet, there remains no sustainable strategy to engage community clinicians in research [20]. Our evaluation data demonstrated that the newly established process codeveloped with CHC providers and practice representatives facilitated several new partnerships with the potential to inform clinical care for a wide range of priority health conditions of importance to those communities.

We recognize that even with these successes, the current process failed to consistently partner researchers and CHCs early in the process, such that continued innovations are necessary. While the development of the research liaison positions along with the virtual learning collaborative was designed to support capacity building for research within the CHCs and the translational science infrastructure, additional resources and long-term organizational commitment will be necessary. These findings are consistent with work by Chinchilla et al which demonstrated that focused capacity building on the relationships between CHCs and translational scientists is a necessary prerequisite to an actual research project partnership [10]. This is further supported by our qualitative data which identified prior relationships and trustworthiness of the researcher as facilitators to partnerships. This is a call to action for translational research program leaders: significant investments in capacity-building programs are essential to support relationship building as a prerequisite to health centers actively engaging in research.

Our evaluation data also demonstrated wide variability in CHC participation in the process and overall acceptance of research requests. While 9 of the 11 affiliated CHC sites chose to actively participate in the newly formed BHN Research Collaborative in 2021, only six were able to fully participate in the learning collaborative throughout the entire course of the 2-year evaluation period. While our qualitative data suggested partnership decisions were largely based on alignment with predetermined rules of engagement, which our Toolkit was designed to support, it is also likely that there were other unmeasured differences across CHCs such as population served, staffing ratios, faculty experience with research, and/or existing relationships with other academic medical centers. National work by Beeson and colleagues demonstrated, for example, that health centers with no previous research experience reported higher percentages of barriers in nearly all categories, compared with health centers that have participated in research activities before [6]. Taken together, these findings suggest a more equitable approach to partnership capacity building is prudent to achieve our goal of greater engagement overall. It is important to note that our qualitative data also found that many partnership decisions were driven by the lack of communication back from researchers through the automated process, namely email. This suggests that our tool was inadequate to facilitate the necessary bidirectional communication for partnership decision-making; and further capacity building with translational scientists is necessary to improve their competency in communication and engagement with diverse groups.

Our novel program evaluation is not without limitations, as the findings only account for a single health system in an urban academic environment with significant NIH funding support. Our evaluation data relied upon input from research liaisons such that partnership status outcomes were incomplete as much as 70% at certain CHCs. We captured missing data via email correspondence and during learning collaborative meetings by sharing CHC-specific data reports. Finally, CHCs do not have exclusive relationships with one academic medical center, so they may be actively partnering with other research programs. Our process did not account for researchers who approached health centers directly, and thus, we are underreporting active research partnerships across the network.

While the BHN Research Collaborative provides the support necessary to initiate a potential research partnership, it is not sufficient to overcome additional barriers to engaging with CHC partners. CHCs identified protected time as a major barrier to recruiting for the RL role at their sites. Staff at health centers already have heavy workloads. While the additional responsibilities were subsidized with a $10000 stipend, this was not sufficient to offset lost clinical revenue for provider time. This is a significant hurdle that will need to be addressed in future work.

A multi-level, iterative approach which builds capacity at the CHC and with translational researchers is necessary to fully address the known barriers to CHCs being active research partners [21–22]. Future opportunities to make continued progress include ongoing fiscal investments, training and education for CHCs and researchers, workforce development, addressing inequitable reimbursement models, and engaging leadership in innovative solutions. Future research should focus on different fiscal partnerships and reimbursement models to ensure CHCs have the resources necessary to support research infrastructure within their practices and demonstrate some measures of return on investment for their participation in research. In this way, academic medical centers and research-intensive institutions could leverage their extensive resources to support CHCs to achieve their full potential as equitable research partners.

**Figure 2.**
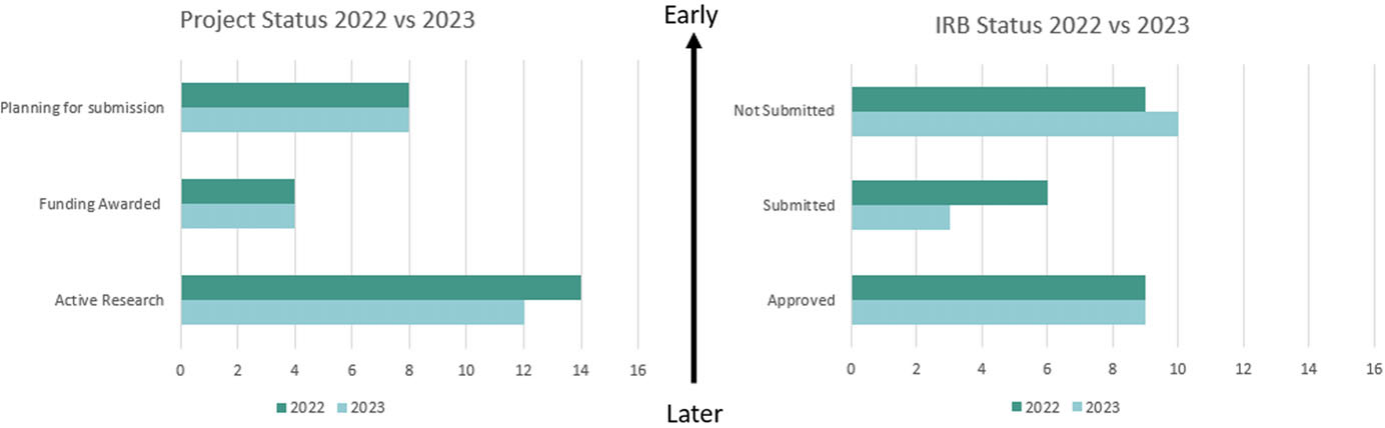
Phase of study lifecycle at the time of research partnership requests: project status and IRB status in 2022 and 2023 (n = 50). *n* = number of individual research projects submitted through the online partnership request form. displays the phase of the study lifecycle at the time of research request for applications submitted in 2022 and 2023, as defined by the project and IRB status. We define project status as where someone is in the cycle of their research projects (i.e. planning for submission, funding awarded but activities not started, or actively conducting research. IRB status entails where a researcher is in the IRB review process (not yet submitted, submitted but awaiting a decision, or approved). The arrow from later to early is a visual representation of how early in the research process a researcher engages with the BHN to form a partnership with a community health center. By either metric and across both years, only about 1/3 of applications were in the early stage (16 out of 50 for project status and 19 out of 50 for IRB status), with early engagement defined by project status = planning and IRB status = not submitted.

**Figure 3.**
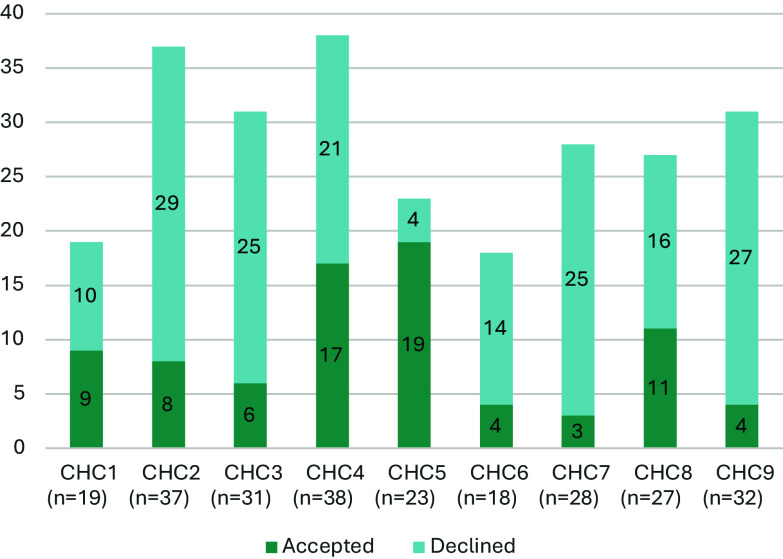
Partnership status of individual research requests across participating CHCs (n = 252). *n* = number of partnership requests sent to each individual health center. CHC = community health center displays the partnership status of these individual research requests across each of the participating CHCs. CHCs 1–9 correspond to the nine individual CHCs that participated in the Boston HealthNet Research Collaborative. As shown, there was variability in the number of research requests received by each CHC (ranging from 18 to 38 requests) and in their respective acceptance rates. For example, CHC7 had the lowest acceptance rate at 11% (3 out of 28 requests), while CHC5 had the highest acceptance rate at 83% (19 out of 23 requests). Of the 252 individual CHC requests that were generated from the 50 studies, 81 (32%) resulted in an acceptance.

## Supporting information

10.1017/cts.2025.10058.sm001 Battaglia et al. supplementary material Battaglia et al. supplementary material

## Supplementary material

For supplementary material/s referred to in this article, please visit https://doi.org/10.1017/cts.2025.10058
