# Supplementary material for: Assessing a community health center-driven process for engaging with translational scientists: What will it take?
Source: J Clin Transl Sci. 2025 Jun 9;9(1):e177. doi: 10.1017/cts.2025.10058 (PMC12444707; doi:10.1017/cts.2025.10058)
Supplement: Battaglia et al. supplementary material [file S2059866125100587sup001.docx]

Supplemental Figure 1: Online Partnership Request Form

Legend

xxx = redacted information

CHC = community health center

CDW = Clinical Data Warehouse

* = required question

**Note:** All the questions in this survey are not necessarily asked to any given researcher. This survey includes logic models to frame questions for particular health centers, in cases where they have asked for specific information to help them make a partnership decision. This is a demonstration of our commitment to make sure the partnership process best suits each health center’s individual needs.

BHN Research Project Request Form

Start of Block: Intro

**Introduction**

This form is to be completed by those who are interested in engaging Boston HealthNet Health Centers in research activities. The intent is to provide relevant information, necessary to help Health Centers determine if the project aligns with their priorities and if partnership is feasible at this time. Please refer to the *Guide for Partnering with BHN CHCs in Research* with the full instructions, prior to submitting this form.

End of Block: Intro

Start of Block: General

**General Information**
*****requires response

Q1 Principal Investigator(s) *****

________________________________________________________________

Q2 Primary contact name (if different than PI)

________________________________________________________________

Q3 Primary contact email *****

________________________________________________________________

Q4 Select the school/college and enter PI's department. Write NONE if there is no department. *****

- Boston Medical Center

__________________________________________________

- BU School of Dental Medicine

__________________________________________________

- BU School of Medicine

__________________________________________________

- BU School of Public Health __________________________________________________
- BU School of Social Work

__________________________________________________

- BU Sargent College

__________________________________________________

- Other

__________________________________________________

Q5 Project title *****

________________________________________________________________

________________________________________________________________

________________________________________________________________

________________________________________________________________

________________________________________________________________

Q6 Sponsor/funding source, fill in grant series or name

- NIH (4) __________________________________________________
- Foundation (5)
- Pharma (6)
- State grant (9)
- Other: (10) __________________________________________________

Q7 IRB of review

________________________________________________________________

Q8 IRB submission status *****

- Not submitted
- Submitted, waiting for approval
- Approved
- Other: __________________________________________________

Q9 BU IRB # (if applicable)

________________________________________________________________

Q10 Award amount (if applicable)

- Total Award amount __________________________________________________
- Estimated amount for CHCs ____________________________________________

Q11 Estimated project timeline *****

________________________________________________________________

________________________________________________________________

________________________________________________________________

________________________________________________________________

________________________________________________________________

Q12 Which health center(s) are you interested in partnering with? (select all that apply) *****
Please indicate the reason you're interested in working with this health center

- CHC 1

__________________________________________________

- CHC 2

__________________________________________________

- CHC 3

__________________________________________________

- CHC 4

__________________________________________________

- CHC 5

__________________________________________________

- CHC 6

__________________________________________________

- CHC 7

__________________________________________________

- CHC 8

__________________________________________________

- CHC 9

__________________________________________________

Q13 Have you had any contact/discussion with Clinical Staff at the Health Center(s) regarding this project? If yes, who and when?

________________________________________________________________

________________________________________________________________

________________________________________________________________

________________________________________________________________

________________________________________________________________

End of Block: General

Start of Block: Project summary

**Project Summary**

Q14 Project Summary (300 word in lay language, copied from IRB application if available) *****
Please include: key research question(s) and description of study population

________________________________________________________________

________________________________________________________________

________________________________________________________________

________________________________________________________________

________________________________________________________________

Q15 Explain how this project supports the Healthy People 2030 national strategy:

________________________________________________________________

________________________________________________________________

________________________________________________________________

________________________________________________________________

________________________________________________________________

Q16 You selected xxx health center, please describe the relevance of your study to xxx population and any plans for xxx-specific analyses or design considerations

________________________________________________________________

________________________________________________________________

________________________________________________________________

________________________________________________________________

________________________________________________________________

End of Block: Project summary

Start of Block: Project Details

**Project Details**

Q17 Which category does your request fall under? *****

- Planning for grant submission and would like to partner with a BHN CHC
- Funding awarded, but project has not started and would like to partner with a BHN CHC
- Active research project in which you would like to add an addendum to include a BHN CHC
- Data only: Request for CHC data use in active research study (to be abstracted from the CDW)
- Data only: Request for CHC data use in feasibility analysis (to be abstracted from the CDW)

Q18 Summary of approach and flexibility of the study methodology (elements that are fixed v. what can be modified) *****

________________________________________________________________

________________________________________________________________

________________________________________________________________

________________________________________________________________

________________________________________________________________

Q19 What are your expectations for CHC staff and patients in this proposed collaboration? *****

________________________________________________________________

________________________________________________________________

________________________________________________________________

________________________________________________________________

________________________________________________________________

Q20 Short explanation of how the study will be implemented at the health center? *****

________________________________________________________________

________________________________________________________________

________________________________________________________________

________________________________________________________________

________________________________________________________________

Q21 What are the benefits and risks to participants (What is the value for our clients to participate) *****

________________________________________________________________

________________________________________________________________

________________________________________________________________

________________________________________________________________

________________________________________________________________

Q22 Which language(s) will you support? Select all that apply *****

- Arabic
- Albanian
- Chinese
- English
- French
- Greek
- Haitian Creole
- Portuguese
- Spanish
- Vietnamese
- Other: __________________________________________________

Q23 How will participants be recognized for their participation? *****

- No recognition
- Financial stipend/incentive (check, gift card, ClinCard, etc)
- Other: __________________________________________________

Q24 How much will participants be compensated? *****

- $1-10 (1)
- $11-25 (2)
- $26-40 (3)
- $41-50 (4)
- $51 or more (5)

Q25 Are there any financial considerations, reimbursements or other benefits built into this study for the CHC? Explanation of financial resources available to the organization to off-set expenses incurred in supporting this project: (What is the value to the organization? What will the organization gain from this?) *****

________________________________________________________________

________________________________________________________________

________________________________________________________________

________________________________________________________________

________________________________________________________________

Q26 Resources required from the organization (How will the health center be involved in the project? Staff engagement/time? Materials/supplies?) *****

________________________________________________________________

________________________________________________________________

________________________________________________________________

________________________________________________________________

________________________________________________________________

Q27 If there is electronic health data from the center involved in your study, please describe what data will be extracted (including personal health (PHI), where the data is being extracted from and how the privacy and data security will be protected or you may attach a copy of the IRB proposal HIPAA form and data security plan

________________________________________________________________

________________________________________________________________

________________________________________________________________

________________________________________________________________

________________________________________________________________

Q28 How will CHC staff be included in sharing/research findings? *****

________________________________________________________________

________________________________________________________________

________________________________________________________________

________________________________________________________________

________________________________________________________________

Q29 Have you submitted a request to the CDW? *****

- Yes
- No

Q30 Provide a brief description of the data you are requesting from the CDW *****

________________________________________________________________

________________________________________________________________

________________________________________________________________

________________________________________________________________

________________________________________________________________

Q31 List specific data fields you are requesting from the CDW *****

________________________________________________________________

________________________________________________________________

________________________________________________________________

________________________________________________________________

________________________________________________________________

**This is the end of the application. Please review all your answers before pressing next. You will not be able to return to the survey after pressing next.**

End of Block: Project Details
